# Knowledge, Attitude, and Practice Toward Diabetic Retinopathy Among People With Diabetes: A Systematic Review and Meta‐Analysis

**DOI:** 10.1002/hsr2.72097

**Published:** 2026-04-16

**Authors:** Parviz Marouzi, Seyyedeh Fatemeh Mousavi Baigi, Firouzeh Fereydouni, Zahra Hemmatian Rabbani, Batoul Haghighi, Reyhaneh Shariati Moghaddam

**Affiliations:** ^1^ School of Paramedical Sciences Mashhad University of Medical Science Mashhad Iran; ^2^ Department of Health Information Technology, School of Paramedical and Rehabilitation Sciences Mashhad University of Medical Sciences Mashhad Iran; ^3^ Student Research Committee Mashhad University of Medical Sciences Mashhad Iran

**Keywords:** attitude, diabetic retinopathy, knowledge, meta‐analysis, practice, systematic review

## Abstract

**Purpose:**

This systematic review and meta‐analysis aimed to evaluate the levels of Knowledge, Attitude, and Practice (KAP) regarding Diabetic Retinopathy (DR) among people with diabetes and to explore demographic and contextual factors influencing these components.

**Methods:**

Following the PRISMA guidelines, we conducted a comprehensive search of PubMed, ScienceDirect, Cochrane Library, and Google Scholar, with no time restriction (updated to 2025). We included 29 questionnaire‐based cross‐sectional studies comprising 14,830 participants. Study quality was assessed using the AXIS tool. Data were analyzed using Comprehensive Meta‐Analysis (CMA) and OpenMeta [Analyst] software with a random‐effects model.

**Results:**

The pooled prevalence of good knowledge, positive attitude, and good practice toward DR was 43.1%, 49.6%, and 40.0%, respectively. Subgroup analysis revealed higher KAP levels in high‐income countries compared to low‐ and middle‐income countries. Meta‐regression showed a statistically significant but minimal effect of positive attitude on good practice (*p* < 0.001), while knowledge was not significantly associated with attitude (*p* = 0.737) or practice (*p* = 0.190).

**Conclusion:**

KAP levels regarding DR remain suboptimal globally. Tailored, context‐specific interventions are essential to improve awareness and preventive behaviors, especially in resource‐limited settings.

## 1 Introduction

Diabetes mellitus (DM) has emerged as one of the most pressing global health challenges, contributing substantially to morbidity, disability, and economic burden worldwide [1]. According to the World Health Organization (WHO), approximately 422 million people were living with DM in 2014—a figure projected to rise to 592 million by 2035 and 700 million by 2045 [2, 3, 4]. The Middle East and North Africa (MENA) region reported the highest global prevalence in 2019 at 12.2%, with estimates suggesting a 96% increase by 2045 [4]. Diabetes‐related complications not only reduce patients' quality of life but also impose significant costs on healthcare systems and societies at large [5, 6].

One of the most serious complications of DM is diabetic retinopathy (DR), a microvascular condition that affects up to 35% of individuals with diabetes and represents a major cause of preventable blindness [1, 7, 8]. In 2020, an estimated 103 million adults were affected by DR worldwide, with this number expected to increase to 160 million by 2045 [7]. The disease progresses silently and often remains asymptomatic until irreversible visual damage occurs [1, 9]. DR is estimated to account for 4.8% of global blindness, affecting over 4 million individuals globally [6, 10].

Preventing vision loss due to DR requires more than clinical screening; it demands active patient participation and health‐seeking behavior. In this context, the Knowledge, Attitude, and Practice (KAP) model provides a behavioral framework for understanding how awareness and beliefs shape health‐related actions [11]. According to this model, increased knowledge can foster positive attitudes, which in turn may promote appropriate health practices. However, KAP levels related to DR vary widely across populations and are influenced by sociodemographic factors such as age, gender, education, income, and occupation [1, 5, 6, 9, 12, 13, 14, 15, 16, 17, 18, 19, 20, 21, 22, 23, 24, 25, 26, 27, 28, 29].

Importantly, research increasingly highlights a disconnect between knowledge and practice, where awareness of DR does not consistently translate into preventive behaviors [5, 6, 15, 23]. This knowledge–practice gap may stem from systemic barriers, cultural beliefs, limited health literacy, or unequal access to healthcare services—particularly in low‐resource settings.

Given the growing burden of DR and the wide variability in KAP levels across populations, a comprehensive synthesis of existing evidence is essential. Therefore, this systematic review and meta‐analysis aims to:

1. Estimate the pooled prevalence of knowledge, attitude, and practice regarding DR among diabetic individuals.
2. Explore how demographic and contextual factors influence these outcomes.
3. Identify gaps and disparities to inform targeted interventions that improve early detection, disease management, and ultimately reduce the socioeconomic burden associated with DR.

## 2 Materials and Methods

### 2.1 Study Design

This systematic review and meta‐analysis were conducted in accordance with the Preferred Reporting Items for Systematic Reviews and Meta‐Analyses (PRISMA 2020) guidelines. The study was designed based on the PICO framework, wherein the population included diabetic patients, the intervention or exposure involved good knowledge and positive attitude toward DR, the comparator comprised individuals with poor or no knowledge and attitude, and the outcome focused on the adoption of preventive practices regarding DR.

### 2.2 Search Strategy

A comprehensive and unrestricted search strategy was employed across four major databases: PubMed, ScienceDirect, Cochrane Library, and Google Scholar. The search combined both Medical Subject Headings (MeSH) and free‐text terms such as “diabetic retinopathy,” “DR,” “diabetes mellitus,” “DM,” “Knowledge, Attitude, Practice,” “KAP study,” and “KAP toward diabetic retinopathy,” using Boolean operators “AND” and “OR.” The search was last updated in 2025. To ensure completeness, reference lists of all included studies were manually screened by two independent reviewers.

### 2.3 Inclusion and Exclusion Criteria

Studies were eligible for inclusion if they were original, questionnaire‐based, cross‐sectional investigations that assessed at least one component of the KAP framework related to DR among diabetic patients, and if they provided extractable quantitative data. Only English‐language articles were included. Studies were excluded if they were reviews, editorials, conference abstracts, or lacked sufficient data for meta‐analysis. Titles and abstracts were independently screened by two reviewers, followed by full‐text review. Disagreements were resolved through discussion with a third reviewer.

### 2.4 Quality Assessment

The quality of the included studies was assessed using the Critical Appraisal Skills Programme (CASP) checklist for qualitative studies. Only studies scoring more than 11 out of 20 were included in the meta‐analysis, ensuring the inclusion of methodologically robust studies and minimizing the risk of bias in pooled estimates.

### 2.5 Data Extraction

Data extraction was conducted independently by two authors using a standardized form. Extracted data included: first author, publication year, country of origin, income classification (based on World Bank criteria), study design, sample size, mean age of participants, and the reported proportions of patients with good knowledge, positive attitude, and good practice regarding DR. Where possible, information on the questionnaire type and healthcare setting was also collected.

### 2.6 Statistical Analysis

Statistical analyses were conducted using Comprehensive Meta‐Analysis (CMA) software version 3.7z and OpenMeta[Analyst]. A random‐effects model (DerSimonian and Laird method) was applied to account for heterogeneity among studies. Pooled prevalence estimates with 95% confidence intervals (CIs) were calculated separately for each KAP domain. Heterogeneity was assessed using Cochran's Q test, *I*² statistic, and Tau² value.

### 2.7 Subgroup, Meta‐Regression, and Bias Analysis

To further explore variability across studies, subgroup analyses were performed based on the income level of the country (high‐income vs. low‐ and middle‐income countries). Additionally, meta‐regression was used to examine the potential moderating effect of mean participant age on KAP outcomes. Publication bias was assessed through funnel plot inspection and Begg and Mazumdar's rank correlation test. Sensitivity analyses were performed by sequentially excluding individual studies to test the robustness of the findings.

## 3 Results

### 3.1 Article Selection Process

**Figure 1:**
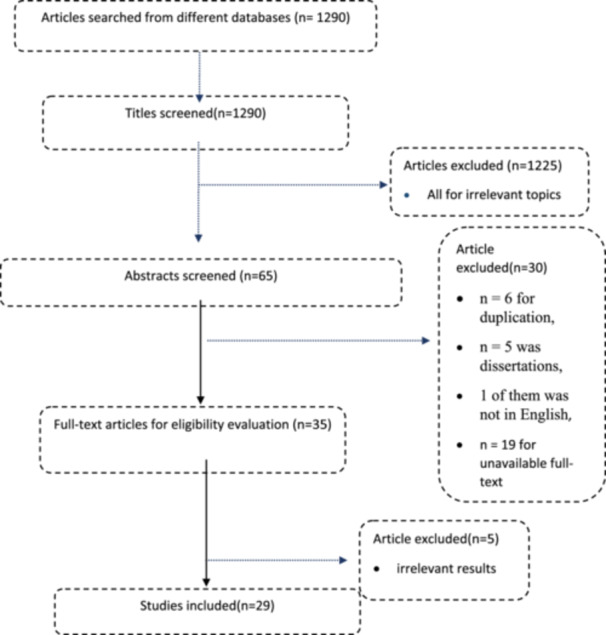
PRISMA flowchart describing the selection of articles.

A total of 1290 articles were initially retrieved from various databases, including PubMed, ScienceDirect, Cochrane Library, and Google Scholar. Following a screening of titles and abstracts, 65 studies were selected for further assessment. After excluding duplicates (6 studies), dissertations (5 studies), non‐English papers (1 study), and those with unavailable full‐texts (19 studies), 29 eligible studies were included in the final analysis. These studies involved a combined sample of 14,830 participants, as detailed in Table 1. The article selection process is illustrated in Figure 1. All included studies were retrospective, descriptive, cross‐sectional, and questionnaire‐based.

**Table 1:** Basic study available information.

Study_ID	Year	N_events	N_total	Country	Mean age	Type of study	Study design
Rani et al. [26]	2008	735	1938	LMIC	29	Original, questionnaire‐based	Qualitative
Al Zarea et al. [14]	2016	332	439	HIC	49.3	Original, questionnaire‐based	Qualitative
Al‐Mulla et al. [9]	2017	255	479	HIC	45.34	Original, questionnaire‐based	Qualitative
Srinivasan et al. [28]	2017	76	288	LMIC	56.64	Original, questionnaire‐based	Qualitative
Alhabdan et al. [6]	2018	1773	2145	HIC	54.14	Original, questionnaire‐based	Qualitative
Phadnis et al. [24]	2018	355	455	LMIC	55.4	Original, questionnaire‐based	Qualitative
AlHargan et al. [16]	2019	215	280	HIC	58.9	Original, questionnaire‐based	Qualitative
Fatima et al. [20]	2019	27	70	LMIC	NA	Original, questionnaire‐based	Qualitative
Gohari et al. [21]	2019	66	732	LMIC	NA	Original, questionnaire‐based	Qualitative
Ali Raza [29]	2019	86	351	LMIC	52	Original, questionnaire‐based	Qualitative
Ahmed et al. [5]	2020	242	623	HIC	46.62	Original, questionnaire‐based	Qualitative
Al‐Asbali et al. [15]	2020	188	200	HIC	52.8	Original, questionnaire‐based	Qualitative
Al‐Yahya et al. [1]	2020	166	313	HIC	52.8	Original, questionnaire‐based	Qualitative
Sharma et al. [27]	2020	215	300	LMIC	50.3	Original, questionnaire‐based	Qualitative
Assem A.S. [30]	2020	110	230	LMIC	49	Original, questionnaire‐based	Qualitative
Abel et al. [12]	2021	70	150	LMIC	55.07	Original, questionnaire‐based	Qualitative
Aly et al. [17]	2021	389	794	LMIC	NA	Original, questionnaire‐based	Qualitative
Farooq et al. [19]	2021	30	72	LMIC	47.5	Original, questionnaire‐based	Qualitative
Javaeed et al. [22]	2021	7	130	LMIC	NA	Original, questionnaire‐based	Qualitative
Zhai et al. [25]	2022	364	1662	LMIC	61.87	Original, questionnaire‐based	Qualitative
Aly A.A. [18].	2022	7	120	HIC	38.201	Original, questionnaire‐based	Qualitative
Keesara [31]	2022	56	196	LMIC	59.89	Original, questionnaire‐based	Qualitative
Ahmed et al. [13]	2023	5	136	LMIC	48.91	Original, questionnaire‐based	Qualitative
Nemivant et al. [23]	2023	69	200	LMIC	64	Original, questionnaire‐based	Qualitative
Eldin Saad N.S. [32]	2023	14	140	LMIC	49.21	Original, questionnaire‐based	Qualitative
Alghahtani T.F. [7]	2023	736	1391	HIC	39.4	Original, questionnaire‐based	Qualitative
R. Bsharat [33]	2024	30	50	LMIC	54	Original, questionnaire‐based	Qualitative
Achar A. [34]	2024	42	82	LMIC	NA	Original, questionnaire‐based	Qualitative
Vasudevan S. [35]	2024	416	500	HIC	39.7	Original, questionnaire‐based	Qualitative

### 3.2 Demographic Characteristics of Participants

The meta‐analysis included 14,830 participants, with a nearly equal gender distribution: 49.24% male and 50.76% female. The mean age of participants was 50.42 ± 8.08 years, with the youngest participant being 18 years old and the oldest being 92 years old. Participants represented a wide range of educational and socioeconomic backgrounds, reflecting a diverse study population. Detailed demographic and clinical characteristics of the study population are presented in Table 2.

**Table 2:** Demographic characteristics of the study population.

Variable		Frequency (%)
Age (years)	< 30 years	23.61
	30–60 years	57.91
	> 60 years	18.48
Gender	Male	49.24
	Female	50.76
Literacy level	Illiterate or primary education	42.80
	Secondary education or high school	29.27
	Post‐graduation	27.84
Economic level	LMIC	43.59
	HIC	56.41
Diabetes duration	< 5 years	28.46
	5–10 years	31.48
	> 10 years	40.06
Type of diabetes	Type I	10.32
	Type II	73.86
	Don't know	15.82

### 3.3 Meta‐Analysis of Knowledge Regarding DR

**Figure 2:**
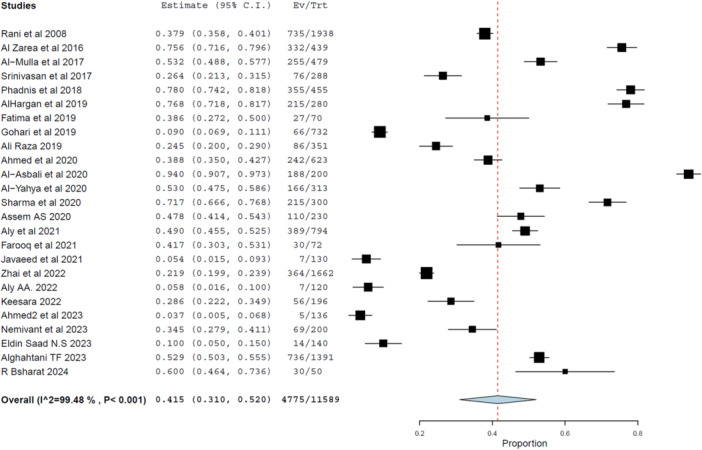
Forest plot illustrating the pooled prevalence of good knowledge about DR among diabetic patients (25 studies). High heterogeneity was observed across studies (*I*² = 99.48%). The heterogeneity assessment showed significant variation across studies (*I*² = 99.48%, Cochran's Q = 4653.10, df = 24, *p* < 0.001), with a Tau² of 0.071, indicating a high level of between‐study variance. These results suggest that knowledge levels differ markedly between populations and settings, underscoring the need for tailored educational interventions.

A total of 25 studies comprising 13,734 diabetic individuals were included to estimate the pooled prevalence of good knowledge about DR. Due to the high variability among studies, a random‐effects model was applied. The pooled prevalence was 41.5% (95% CI: 31.0%–52.0%, *p*< 0.001), as shown in Figure 2.

#### 3.3.1 Subgroup Analysis Based on Country Income Level

Subgroup analysis based on World Bank income classification revealed disparities. The pooled prevalence of good knowledge was 56.3% (95% CI: 37.5%–75.0%) in high‐income countries (HICs) and 34.4% (95% CI: 24.1%–44.8%) in low‐ and middle‐income countries (LMICs), both with significant heterogeneity (*I*² > 99%). These findings emphasize income‐level inequities in DR awareness and the urgent need for equity‐focused health education.

#### 3.3.2 Cumulative and Sensitivity Analyses

Cumulative meta‐analysis indicated that the pooled prevalence remained relatively stable over time at 41.5%, suggesting limited progress in public knowledge despite ongoing global efforts.

Sensitivity analysis confirmed the robustness of findings. When individual studies were sequentially excluded, pooled estimates fluctuated only slightly (range: 40.0%–43.1%), and all results remained statistically significant.

#### 3.3.3 Meta‐Regression: Impact of Mean Age on Knowledge

Meta‐regression analysis revealed that mean age did not significantly influence knowledge levels (coefficient = 0.005; 95% CI: −0.009 to 0.018; *p* = 0.491), suggesting that factors other than age (e.g., education level, access to information) may have a stronger influence.

#### 3.3.4 Publication Bias Assessment

**Figure 3:**
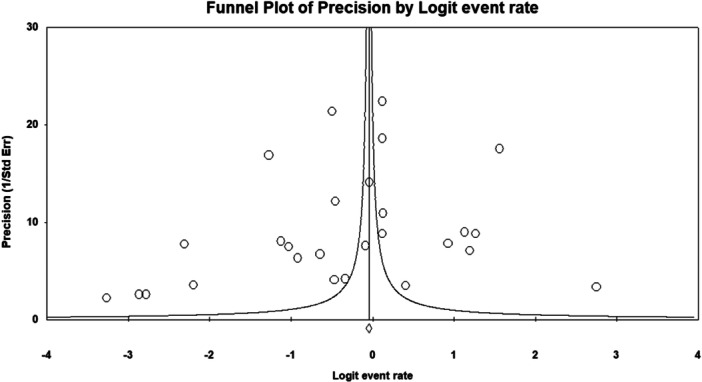
Funnel plot for knowledge‐related studies, showing symmetry and indicating no significant publication bias.

The funnel plot exhibited a symmetrical distribution (Figure 3), and Begg and Mazumdar's test showed no evidence of publication bias (Kendall's tau = −0.145, *p* = 0.287).

### 3.4 Meta‐Analysis of Positive Attitude Toward DR

**Figure 4:**
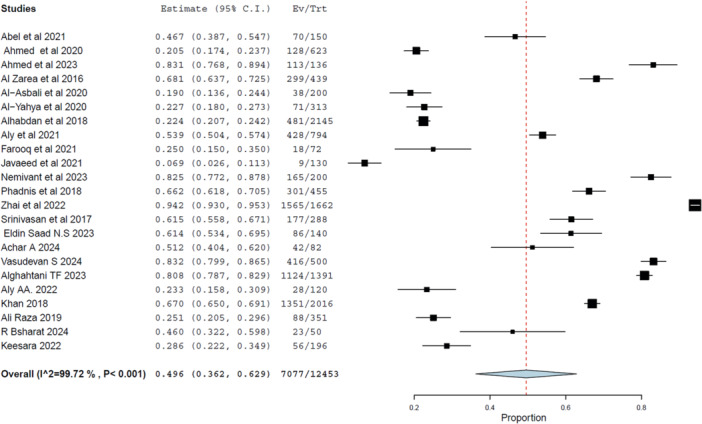
Forest plot displaying pooled prevalence of positive attitude toward DR. Note the significant variability across studies.

Twenty‐two studies involving 12,453 participants reported data on positive attitude toward DR. The pooled prevalence was 49.6% (95% CI: 36.2%–62.9%, *p* < 0.001), as presented in Figure 4.

Substantial heterogeneity was observed (*I*² = 99.7%, *τ*² = 0.105, *p* < 0.001), prompting further exploration of moderating variables such as geography and health system structure.

**Figure 5:**
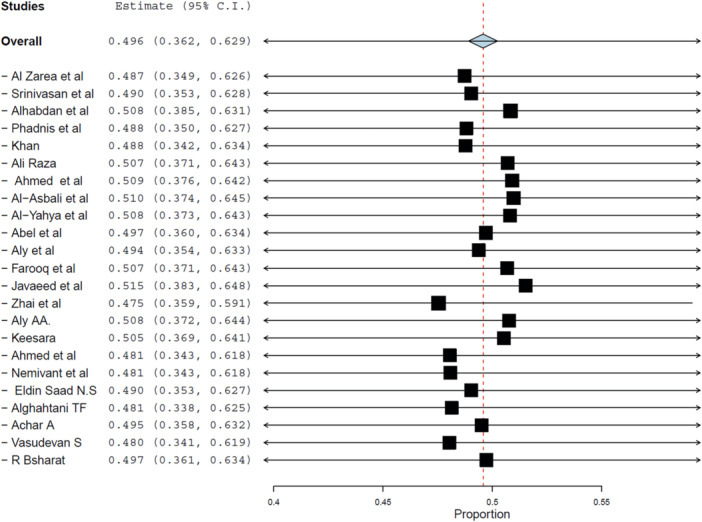
Sensitivity analysis for attitude toward DR. Removal of individual studies did not significantly alter the pooled estimates.

**Figure 6:**
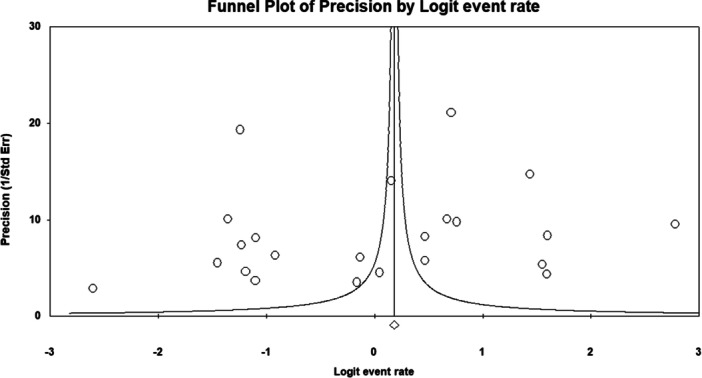
Funnel plot showing symmetrical distribution, indicating absence of publication bias in attitude‐related studies.

Sensitivity analysis supported the robustness of findings (Figure 5), and no evidence of publication bias was identified via funnel plot inspection (Figure 6).

#### 3.4.1 Subgroup Analysis by Income Level

The pooled prevalence of positive attitude was slightly higher in LMICs (52.2%, 95% CI: 32.2%–72.3%) compared to HICs (46.2%, 95% CI: 28.5%–63.8%). Despite this, both subgroups exhibited extreme heterogeneity, reflecting the influence of contextual and cultural factors beyond economic classification.

### 3.5 Meta‐Analysis of Good Practice Toward DR

**Figure 7:**
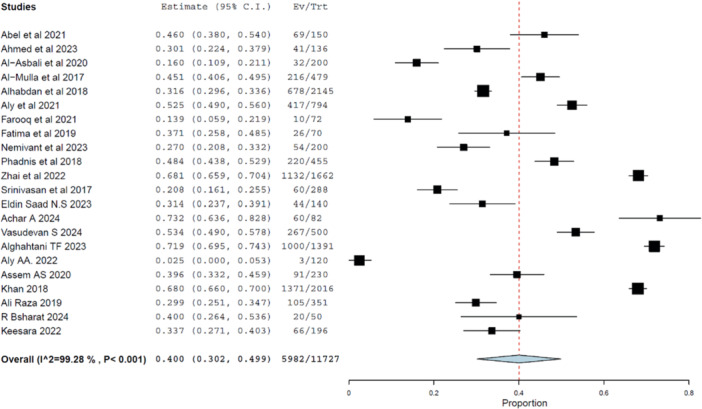
Forest plot showing pooled prevalence of good practice toward DR. Considerable heterogeneity reflects contextual variability in health behavior and system performance.

Twenty‐one studies involving 11,727 diabetic patients were analyzed. The pooled prevalence of good practice was 40.0% (95% CI: 30.2%–49.9%, *p*< 0.001), as shown in Figure 7. Heterogeneity remained high (*I*² = 99.28%).

**Figure 8:**
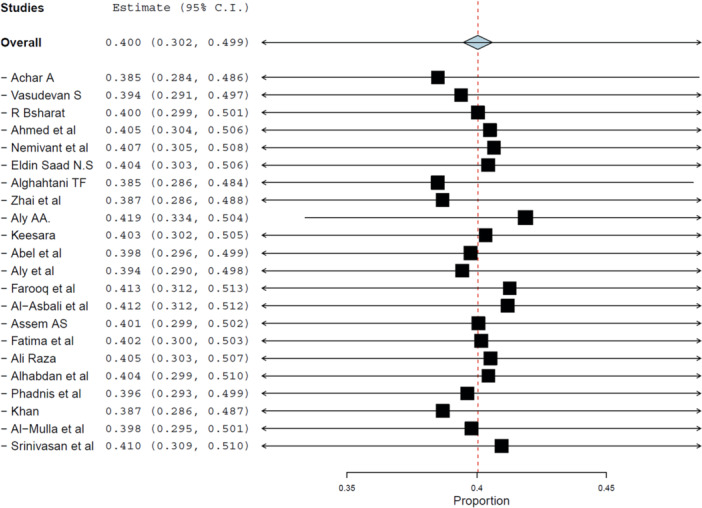
Sensitivity analysis of practice‐related estimates. Results remained stable when studies were sequentially excluded.

**Figure 9:**
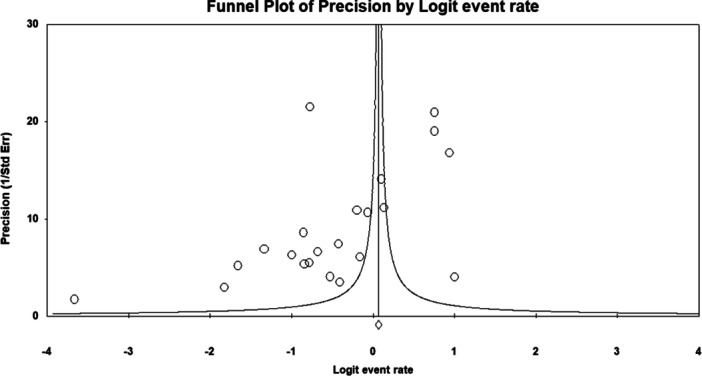
Funnel plot indicating no significant publication bias for practice‐related studies.

Sensitivity analysis confirmed the consistency of findings (Figure 8), and no evidence of publication bias was found (Figure 9).

#### 3.5.1 Subgroup Analysis by Income Level

The prevalence of good practice was 38.5% (95% CI: 27.2%–49.8%) in LMICs and 42.6% (95% CI: 24.6%–60.7%) in HICs, showing slightly better outcomes in wealthier countries. Nonetheless, both groups remained well below optimal adherence thresholds, indicating widespread barriers to practice.

### 3.6 Meta‐Regression: KAP Interrelationships

Meta‐regression analysis showed that good knowledge had no significant effect on good practice (coefficient = 0.000, *p* = 0.190). Similarly, knowledge did not significantly influence attitude (*p* = 0.737). However, a positive attitude had a minimal but statistically significant effect on good practice (coefficient = 0.000, *p* < 0.001).

These findings reveal a clear knowledge–practice gap, which cannot be addressed solely by increasing awareness. Behavioral economics suggests that factors like present bias, cognitive load, inertia, and limited self‐efficacy may prevent individuals from translating knowledge into action. Structural barriers—such as healthcare costs, insurance coverage, transportation access, and waiting times—also inhibit proactive behavior. A summary of the pooled prevalence estimates for good knowledge, attitude, and practice toward DR is presented in Table 3.

**Table 3:** Pooled prevalence estimates for good KAP regarding DR.

Parameters	Frequency (%)	95% CI
Good knowledge	43.1	31.3–54.9
Positive attitude	49.6	36.2–62.9
Good practice	40	30.9–49.9

Furthermore, cultural beliefs and health literacy may moderate the KAP relationship. For instance, in some communities, DR may not be perceived as an urgent threat due to its asymptomatic nature, or fatalistic attitudes may reduce the perceived value of screening. Thus, effective interventions must integrate culturally sensitive messaging, simplify information delivery, and address both behavioral and systemic obstacles.

## 4 Discussion

DR, a leading microvascular complication of DM, continues to be a major cause of visual impairment and blindness, particularly among the working‐age population [1, 19, 25]. Early diagnosis and effective management of DR are vital in preventing irreversible vision loss and reducing associated personal and healthcare system burdens. Given that DR is largely preventable and treatable in its early stages, increasing awareness, fostering patient engagement, and improving preventive behaviors are essential. This systematic review and meta‐analysis synthesized findings from 29 studies to evaluate the levels of KAP toward DR and to examine how demographic and contextual factors shape these outcomes.

### 4.1 Suboptimal Knowledge: A Barrier to Early Action

The pooled prevalence of good knowledge about DR was 43.1% (95% CI: 31.3–54.9), suggesting that fewer than half of individuals with diabetes are adequately informed about this complication. This level of awareness is insufficient, especially considering the critical role of knowledge in driving screening adherence and early intervention [1, 5, 6, 15, 16, 18, 21]. Despite extensive public health campaigns and growing scientific literature, cumulative meta‐analysis revealed no significant improvement in knowledge levels over time. Furthermore, meta‐regression analysis showed no meaningful association between age and knowledge, indicating that broader structural determinants—such as educational attainment, healthcare access, and public health infrastructure—may play more decisive roles.

Income‐based subgroup analysis highlighted significant disparities, with higher knowledge prevalence in high‐income countries (56.3%) compared to LMICs (34.4%). These findings emphasize the unequal distribution of information resources and call for contextualized educational interventions to bridge global health literacy gaps.

### 4.2 Attitudes and the Role of Cultural and Social Contexts

Our analysis showed that nearly half of the participants (49.6%; 95% CI: 36.2–62.9) held positive attitudes toward DR prevention. However, the lack of clear differences between HICs and LMICs suggests that factors beyond income—such as cultural beliefs, trust in the health system, and community engagement—may be more influential [1, 6, 12].

The absence of improvement in attitude levels over time, along with the weak correlation between knowledge and attitude (*p* = 0.737), underscores a disconnect between awareness and mindset transformation. Traditional awareness campaigns may not adequately address cultural norms, language barriers, or belief systems. This highlights the importance of culturally sensitive and community‐based strategies to shift attitudes and enhance motivation for preventive behaviors.

### 4.3 Persistent Gaps in Preventive Practices

Perhaps the most critical insight from this review is the consistently low prevalence of good practices related to DR, which stood at 40.0% (95% CI: 30.2–49.9). These behaviors—such as undergoing regular retinal screening, attending ophthalmic consultations, and adhering to treatment—are vital for preventing visual disability. Alarmingly, practice levels were low even in high‐income countries (42.6%) and slightly lower in LMICs (38.5%), suggesting that awareness alone is insufficient to promote health‐protective actions.

Meta‐regression revealed no significant influence of age or knowledge on practice (*p* = 0.190), indicating that behavioral and systemic barriers are at play. These may include logistical challenges (e.g., transportation, cost, appointment availability), low health literacy, limited family or provider support, and behavioral economics phenomena such as present bias, inertia, and cognitive overload.

### 4.4 Decoupling of KAP Components: Behavioral and Structural Explanations

A core strength of this study is its examination of KAP interrelationships. Our findings showed that while positive attitude had a statistically significant (albeit minimal) effect on practice (*p* < 0.001), knowledge did not significantly influence either attitude or practice. This contradicts the classic linear KAP model and aligns with contemporary behavioral science, which recognizes that knowledge is necessary but not sufficient for behavior change [1, 5, 6, 7, 9, 12, 13, 14, 15, 16, 17, 18, 19, 20, 21, 22, 23, 24, 25, 26, 27, 28, 29].

To bridge the knowledge–practice and attitude–practice gaps, we must consider mediating factors such as provider communication quality, social support, and systemic facilitators like insurance coverage, clinic proximity, and service integration. Future research should apply behavioral frameworks (e.g., COM‐B model, Health Belief Model) and test interventions that reduce structural barriers or leverage nudges to promote action. Additionally, health literacy must be assessed as a moderator in KAP dynamics, especially in underserved or rural populations.

### 4.5 Implications for Public Health Policy and Practice

These findings have significant implications for DR prevention and chronic disease management. Given the low and uneven KAP levels, particularly in LMICs, a multi‐tiered strategy is essential:

- Integrate DR education into routine diabetes management, especially at the primary care level.
- Establish and scale up community‐based screening programs with strong referral and follow‐up mechanisms.
- Implement mass communication campaigns using local languages, culturally adapted content, and social media platforms.
- Train general practitioners and primary care staff to assess and enhance patient KAP during consultations.
- Evaluate and improve healthcare providers' own knowledge and attitudes toward DR.

Furthermore, the use of digital innovations holds promise. Artificial intelligence (AI) can tailor educational content, send screening reminders, and support clinical decision‐making. Virtual reality (VR) tools may improve patient engagement through immersive education. Blended learning platforms and mobile health (mHealth) solutions can overcome literacy and accessibility barriers, particularly in remote regions [36, 37, 38, 39, 40, 41].

Despite the robustness of this meta‐analysis, a key limitation lies in the lack of individual‐level data. Future individual participant data (IPD) meta‐analyses are needed to explore effect modifiers such as gender, comorbidities, or socioeconomic status and design more precise interventions.

## 5 Conclusion

This systematic review and meta‐analysis highlight persistent and widespread gaps in KAP related to DR among individuals with diabetes—particularly in LMICs. While patients in HICs demonstrate relatively better knowledge and practice, attitude levels remain comparably low across economic contexts. Notably, the weak associations between knowledge, attitude, and actual preventive behavior emphasize that awareness alone is insufficient to drive effective DR management.

The lack of progress in KAP indicators over time underscores the urgent need for targeted, context‐specific interventions that address both behavioral and structural barriers. Strategies such as integrating DR education into routine diabetes care, expanding accessible and community‐driven screening programs, and strengthening the role of primary care providers are critical to improving early detection and preventing vision loss. Future research should move beyond aggregate data to explore individual‐level behavioral determinants, such as cultural beliefs, health literacy, and socioeconomic constraints. Advanced approaches—including behavioral science frameworks, digital health tools, and equity‐oriented public health strategies—will be essential to close the KAP gaps and promote sustainable improvements in DR prevention and management.

## Author Contributions

Conception and design of study: Firouzeh Fereydounia and Zahra Hemmatian Rabbani. Data collection: Firouzeh Fereydounia, Zahra Hemmatian Rabbani, Batoul Haghighi, Reyhaneh Shariati Moghaddam. Analysis and interpretation of results and revising manuscript: Seyyedeh Fatemeh Mousavi Baigib and Parviz Marouzia. Drafting the manuscript: Firouzeh Fereydounia and Seyyedeh Fatemeh Mousavi Baigib. All authors reviewed the results and approved the final version of the manuscript.

## Funding

The authors have nothing to report.

## Ethics Statement

This work was approved by the ethics committee of Mashhad University of Medical Sciences (authorization number: IR.MUMS.FHMPM.REC.1402.242).

## Conflicts of Interest

The authors declare no conflicts of interest.

## Transparency Statement

The corresponding author, Firouzeh Fereydouni, affirms that this manuscript is an honest, accurate, and transparent account of the study being reported; that no important aspects of the study have been omitted; and that any discrepancies from the study as planned (and, if relevant, registered) have been explained.

## Data Availability

Data can be obtained from the writers when asked.
